# In this issue of *Current Oncology*

**Published:** 2006-10

**Authors:** M. McLean

Beginning with this issue, which coincides with Breast Cancer Awareness Month, *Current Oncology* is pleased to announce its association with Eli Lilly’s Oncology on Canvas. The Eli Lilly Oncology on Canvas International Art Competition and Exhibition provides people affected by cancer with the opportunity to express visually and in narrative form the life-affirming changes that have given their journey meaning. The competition invites people who have been diagnosed with cancer, their oncologists, nurses, family members, and caregivers, and artists and art students to share their cancer journey with the world through art. Further information about this initiative can be found at www.lillyoncology.com/oncology_canvas/2006_index.jsp?reqNavId=5.1.

Selected pieces from the exhibition will be published on the front cover of *Current Oncology* starting with this issue and continuing throughout 2007. The accompanying artist’s narrative will be published on the issue’s contents page, together with information about the contest.

To further mark Breast Cancer Awareness Month, we include a timely review from Dr. Sunil Verma and Dr. Paula Fishman, outlining recent advances in the field of breast cancer, with particular reference to systemic adjuvant therapy and upcoming studies in this changing field.

In the next issue, our Ethics Editor, Dr. Mark Handelman, will be presenting a submission—“The Cancer Patient’s Wife”—that uses a slightly different format to explore important moral, legal, and ethical issues surrounding the terminally ill patient and the process of completing “do not resuscitate” orders. We hope that this article will generate dialog with our readers.

I would also like to take time to thank others in our dedicated group of editors. In next issue, Dr. Richard Mould is launching a History of Oncology series, beginning with a commemoration of the death of Pierre Curie (1859–1906); Dr. Steven Sagar handles Integrative Therapies for Oncology; Dr. Jan Seuntjens oversees Medical Physics; Dr. Richard Ablin covers Updates and Developments in Oncology; Dr. Fred Saad manages submissions on Surgical Oncology; and Dr. Teri Langlois looks after Nutrition. With the help of our subeditors and the generous support of industry through their advertising budgets and educational grants, these colleagues have brought about a significant increase in manuscript submissions and allowed *Current Oncology* to move to a six-issue publication schedule this year.

